# Conformational modulation of intrinsically disordered transactivation domains for cancer therapy

**DOI:** 10.1093/pnasnexus/pgaf152

**Published:** 2025-05-09

**Authors:** Thibault Vosselman, Cagla Sahin, David P Lane, Marie Arsenian Henriksson, Michael Landreh, Dilraj Lama

**Affiliations:** Department of Microbiology, Tumor and Cell Biology (MTC), Karolinska Institutet, Biomedicum, Solnavägen 9, SE-171 65 Stockholm, Sweden; Department of Microbiology, Tumor and Cell Biology (MTC), Karolinska Institutet, Biomedicum, Solnavägen 9, SE-171 65 Stockholm, Sweden; Structural Biology and NMR Laboratory and the Linderstrøm-Lang Centre for Protein Science, Department of Biology, University of Copenhagen, Ole Maaløes Vej 5, 2200 Copenhagen, Denmark; Department of Microbiology, Tumor and Cell Biology (MTC), Karolinska Institutet, Biomedicum, Solnavägen 9, SE-171 65 Stockholm, Sweden; Department of Microbiology, Tumor and Cell Biology (MTC), Karolinska Institutet, Biomedicum, Solnavägen 9, SE-171 65 Stockholm, Sweden; Department of Microbiology, Tumor and Cell Biology (MTC), Karolinska Institutet, Biomedicum, Solnavägen 9, SE-171 65 Stockholm, Sweden; Department of Cell and Molecular Biology, Uppsala University, Husargatan 3, SE-751 24 Uppsala, Sweden; Department of Microbiology, Tumor and Cell Biology (MTC), Karolinska Institutet, Biomedicum, Solnavägen 9, SE-171 65 Stockholm, Sweden

**Keywords:** disorder, transactivation domain, conformational modulation, cancer

## Abstract

Intrinsically disordered proteins are implicated in many diseases, but their overrepresentation among transcription factors, whose deregulation can cause disproportionate expression of oncogenes, suggests an important role in cancer. Targeting disordered transcription factors for therapy is considered challenging, as they undergo dynamic transitions and exist as an ensemble of interconverting states. This enables them to interact with multiple downstream partners, often through their transactivation domains (TADs) by the mechanisms of conformational selection, folding-upon-binding, or formation of “fuzzy” complexes. The TAD interfaces, despite falling outside of what is considered “classical” binding pockets, can be conformationally modulated to interfere with their target recruitment and hence represent potentially druggable sites. Here, we discuss the structure–activity relationship of TADs from p53, c-MYC, and the androgen receptor, and the progresses made in modulating their interactions with small molecules. These recent advances highlight the potential of targeting these so far “undruggable” proteins for cancer therapy.

## Introduction

Today, we have a good understanding of how different cancers originate and a wide array of treatment options at our disposal, yet many cancers are still marked by poor prognosis for patient survival due to their intricate biology with high complexity. Lack of early detection tools and absence of efficient treatment often form the foundation for these cases. As a result, cancers consistently rank among the main causes of death worldwide, accounting for ∼30% of all mortalities due to noncommunicable diseases (1). Interestingly, it is estimated that a significant proportion (∼70%) of the proteins that are linked to aberrant cellular control in cancer are disordered, either in specific regions or over their entire sequence (2). This class of proteins termed intrinsically disordered proteins (IDPs) are ubiquitous and play pivotal roles in regulating a wide range of cellular processes (3). The recent progress made in understanding the molecular basis of their involvement in driving tumor initiation, progression, and metastasis has made them a major interest for cancer drug development efforts (2–5).

IDPs lack a stable tertiary structure and hence do not contain a defined 3D conformation under physiological conditions. The absence of structural definition renders them highly dynamic and allows these proteins to undergo rapid transitions between various structural states in response to environmental cues or molecular interactions (6). Their flexibility enables IDPs to engage in specific yet transient interactions with a wide variety of binding partners. They also exhibit a heterogeneous dispersal of structural conformations when not in direct proximity to protein interactors. In extension, their conformational flexibility allows IDPs to be highly adaptive to changes in the cellular environment and molecular modifications (7). Logically, this “shape-shifting” ability makes IDPs extremely challenging to target through conventional structure-based drug-design approaches that require the presence of a stable binding site, and therefore often have been considered to be undruggable (2–5). It is, however, a compelling avenue for exploration as the proteome space for therapeutic interventions will be dramatically widened when the disordered class of proteins can be rationally targeted. Hence, strategies for the discovery or design of small molecules that can specifically modulate their conformational landscape offer exciting possibilities for drug development. Here, we discuss how the structural flexibility and protein binding activity of disordered transactivation domains (TADs) present in transcription factors can be modulated using small molecules to target cancer.

## Disordered TADs in cancer

Transcription factors regulate gene expression by recognizing specific DNA sequences and forming multiprotein complexes. Their overactivation or deregulation can lead to disproportionate transcription of genes involved in the development of tumors, making them promising candidates for the development of therapeutics (8–10). Within the modular architecture of a transcription factor, disordered regions act as flexible scaffolds that contain binding sites for other proteins, which stimulate the activation of associated genes. These domains, commonly referred to as TADs, can engage in a multitude of interactions with different protein binders (9). The intrinsic disorder of the TAD is therefore a common and indispensable structural property that regulates the biological activity of cancer-related and targeted transcription factors such as p53, c-MYC, and the androgen receptor (AR).

### p53

**Fig. 1.**
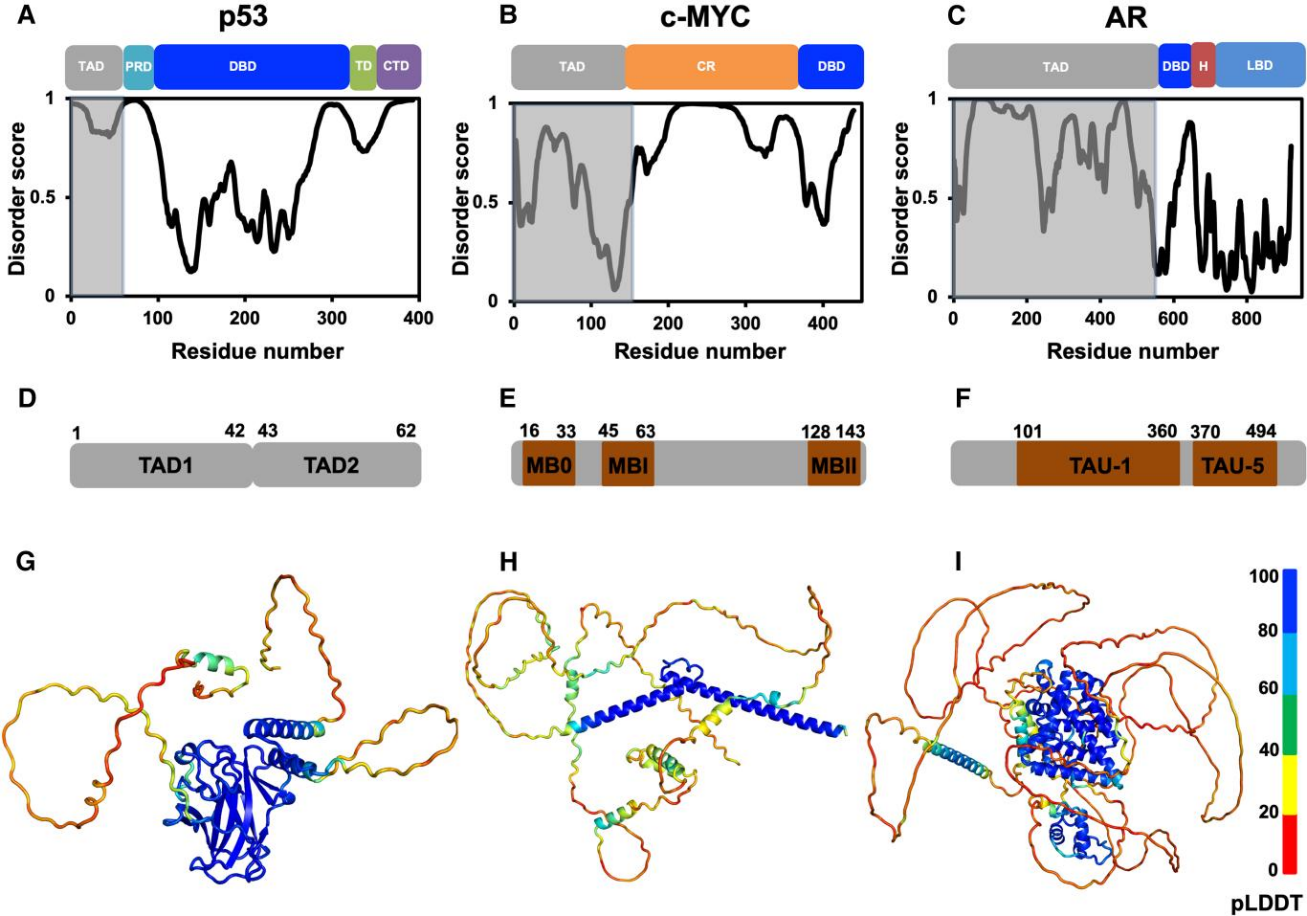
Domains, disorder, TADs, and structure models. A)–C) First row: domain architecture of p53 (TAD, transactivation domain; PRD, proline-rich domain; DBD, DNA-binding domain; TD, tetramerization domain; CTD, C-terminal domain), c-MYC (TAD, transactivation domain; CR, central region; DBD, DNA-binding domain), and AR (TAD, transactivation domain; DBD, DNA-binding domain; H, hinge; LBD, ligand-binding domain), respectively. Second row: sequence-based disorder prediction generated using the VSL2 algorithm from the Predictor of Natural Disorder Regions (PONDR) server is shown below each protein. The TAD regions are highlighted in gray background on each of the plots. D)–F) Subdomains (TAD1 and TAD2), functional motifs (MB0, MBI, and MBII), and transcription units (TAU-1 and TAU-5) of the TAD from p53, c-MYC, and AR, respectively. The residue range of the subdomains, MB motifs, and TAU units are indicated. G)–I) AlphaFold-generated models of full-length p53 (UniProt ID: P04637), c-MYC (UniProt ID: P01106), and AR (UniProt ID: P10275), respectively. The residues in the modeled structures are color-coded based on their predicted local distance difference test (pLDDT) scores, whose range from low to high is considered as a good predictor of disorder to order in a structure (14,).

The tumor suppressor p53 is one of the most extensively studied and prominent examples of a TAD-containing protein with direct involvement in carcinogenesis (11). It has a crucial role in protecting the genomic stability and as such been described as the “guardian of the genome” (12). The protective effects take place through direct involvement in the cellular response to both internal and external stress signals, including DNA damage (13). p53 is a predominantly disordered protein except for the folded DNA-binding domain and the helical tetramerization domain (Fig. 1A, D, and G). The disordered N-terminal region contains a TAD followed by a proline-rich domain. The TAD in p53 can be further divided into two subdomains (TAD1 and TAD2) that interact with various transcriptional cofactors, allowing the precise regulation of target gene expression involved in cellular processes such as cell cycle arrest, DNA repair, and apoptosis (11–15).

### c-MYC

The proto-oncoprotein c-MYC is another prominent example of a cancer-related TAD (16). It is a master regulator of gene expression and modulates cellular processes including cell proliferation, differentiation, metabolism, and controlled cell death (17). Unlike p53, c-MYC is a fully disordered protein, and the domain composition can be broadly categorized into an N-terminal TAD, a central region, and a C-terminal DNA-binding domain (Fig. 1B, E, and H). The disordered N-terminal TAD contains three functionally important and evolutionary conserved motifs termed MYC Box 0 (MB0), MYC Box I (MBI), and MYC Box II (MBII). The MYC boxes mediate the recognition of several transcriptional cofactors, which have an important role in gene expression that is quintessential for tumor formation (18, 19). In line with its central role in proliferation, deregulation of c-MYC is a common characteristic across a broad spectrum of human cancers (20).

### AR

The same principle as in p53 and c-MYC can be found in the AR, a member of the nuclear hormone receptor family of transcription factors (21). Deregulation of AR is strongly linked to prostate cancer biology, making it a major therapeutic target in this disease (22, 23). In addition, it is also emerging as an important factor in breast cancer pathology, glioblastoma, and several other types of human malignancies in which AR-signaling facilitates tumor growth (24, 25). This receptor is a multidomain protein with a considerably large intrinsically disordered N-terminal TAD, followed by the folded DNA- and ligand-binding domains, which are separated by a short hinge region (Fig. 1C, F, and I). The TAD contains two transcriptional activation units called TAU-1 and TAU-5 that are responsible for regulating the AR transcriptional activity by recognizing coactivators, corepressors, and driving interdomain interactions (26).

## TADs can engage their protein interactors in diverse modes

**Fig. 2.**
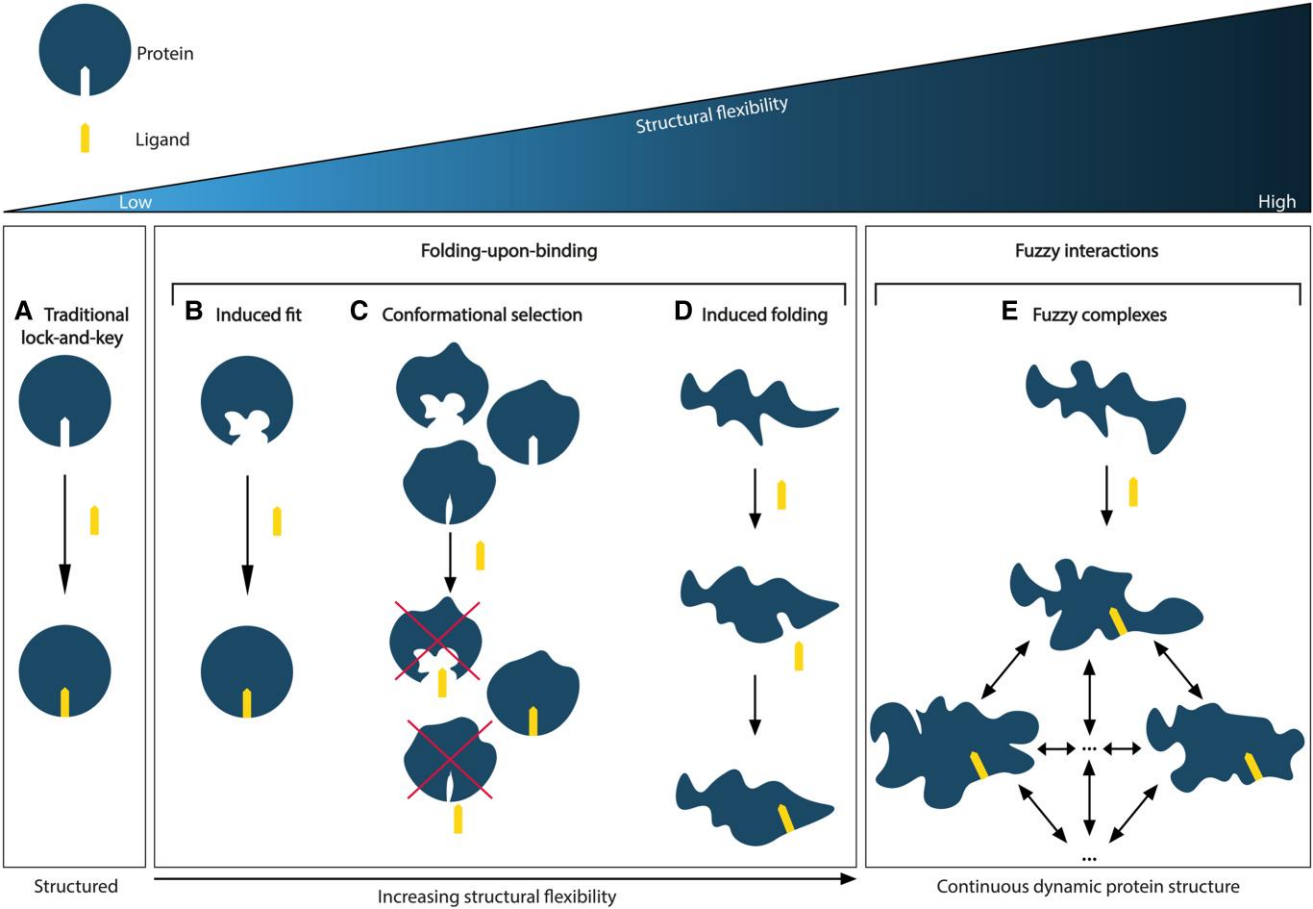
Schematic representation of protein interaction functionality on a rigid-to-dynamic scale. Top row: schematic representation of protein and ligand. Low to high structural flexibility as indicated. A) The traditional “lock-and-key” principle conceptualizes the interaction between protein and ligand where the ligand represents the “key” and the binding site in the protein structure represents the “lock.” B) The induced fit concept displaying structural alteration of the ligand-binding site in the protein structure as response to the binding partner being in its proximity and initiating interaction. C) Conformational selection of the fitting structural conformation of the protein by the binding partner. D) Depiction of induced folding of the dynamic protein structure of IDPs when the disordered protein interacts with its binding partner. E) Fuzzy complex formation of IDPs, the disordered protein structure maintains structural plasticity in final complexes with binding partners.

The classical “lock-and-key” mechanism of interaction can effectively describe the recognition between two relatively rigid and structurally ordered biomolecules (Fig. 2). However, the highly disordered structures of TADs make it challenging to decipher how they specifically recognize and interact with their binding partners. TADs can undergo “folding-upon-binding” and acquire specific structures induced by their binding partners (27–34). They can also adopt multiple interconverting conformations in the bound complex resulting in what is termed as “fuzzy interactions” (35–42). These two models are postulated to be the main mechanisms for how TADs engage with their interactors (Fig. 2).

Folding-upon-binding typically represents acquisition of a well-defined secondary structure by the unstructured polypeptide in response to binding interactions with another molecule (27–30). This disorder-to-order transition offers a window to analyze and characterize the end-state structure of disordered region through traditional structural biology techniques (31). However, the folding-upon-binding model cannot explain when this structural transition occurs. One possibility is that the association is initiated in a fully disordered state and that folding is induced by interaction with the binding partner. This theory is referred to as the “induced folding” approach (27, 32, 33). The “conformational selection” hypothesis, on the contrary, suggests that this conformational change is not initiated by the interaction, but instead that the structural repertoire of disordered polypeptides provides both folded and unfolded conformational states in solution. Binding occurs when the prefolded structural conformations are selected by the binding partner to initiate the interaction (33). A possible third model is an amalgamation of the two theories, where the initial interaction is formed via “conformational selection,” followed by optimization of the bound-state structure through “induced folding” (34).

Fuzzy interactions are defined as interactions in which disordered regions do not adopt well-defined 3D conformations in the bound state, but instead contain a significant amount of heterogeneity. In other words, they maintain their structural plasticity in the final complexes, which is also referred to as “fuzzy complexes” (35). An interesting postulation concerning this type of interaction is whether two IDPs could interact through a fuzzy complex, while both remain partially flexible. Currently, four different types of fuzzy complex models have been described based on the conformational property and dynamics of the interacting segments (36). These include “polymorphic” complexes in which the bound fragment adopts alternative conformations in the complex (37), “clamps” in which a disordered segment serves as linker between two folded elements in the complex (38), “flanking” complexes in which disordered segments provide supplementary contact points for binding (39), and “random” complexes in which varying short-length recognition motifs are connected by multiple disordered segments (40). Despite this categorization, it is important to note that these subtypes of fuzzy interactions are not mutually exclusive but can occur in parallel with each other within the same complex (36).

Fuzzy complexes lack the stable bound conformation and therefore cannot generate the same amount of enthalpy to compensate for the loss in conformational entropy. Thus, they often have lower strengths than complexes formed through folding-upon-binding (41). This can be advantageous in systems where interactions must be transient and reversible to ensure that a signaling cascade is turned on or off, for example, in gene transcription. Another advantage of intrinsic disorder is described via the “fly-casting hypothesis” (42). This model proposes that the extended conformations of unfolded structures like TADs, which have a larger radius than folded domains, enable initial contact with binding partners when they are further away from one another. They then pull their target closer, allowing complex formation to proceed and adopt specific mode of engagements as discussed above.

## Structurally characterized protein interactions of TADs

The disordered nature of TADs makes it challenging to explore their structural properties, and our current knowledge is predominantly derived from bound-state structures of TAD fragments with their binding partners. Such complexes of p53 and c-MYC extracted from the Protein Data Bank (PDB) are listed in Table 1. Further, representative examples from the list are described in the section below, which highlight the heterogeneous nature of their bound conformations and how they can be exploited for therapeutic interventions. It is interesting to note that thus far, no structure of TAD from the AR with its binding partners has been experimentally determined.

**Table 1.** Structures of the p53 and c-MYC TADs in complex with protein binders.

PDB ID	TAD residue range^a^	Protein binders	Method^b^	References
**p53**				
1YCR	17–29	MDM2	X-ray	(43)
1YCQ	17–27	MDM2	X-ray	(43)
3DAC	17–28	MDMX	X-ray	(44)
3DAB	18–28	MDMX	X-ray	(44)
6T58	17–30, 36–54	S100A4	X-ray	(45)
2B3G	33–56	Replication protein A	X-ray	(46)
2K8F	1–39	p300	NMR	(47)
2MZD	35–59	p300	NMR	(48)
2LY4	14–60	High mobility group B1	NMR	(49)
5HOU	1–60	CREB-binding protein	NMR	(50)
5HPD	2–61	CREB-binding protein	NMR	(50)
5HP0	37–61	CREB-binding protein	NMR	(50)
2L14	13–61	CREB-binding protein	NMR	(51)
2GS0	45–58	TFB1 subunit of TFIIH	NMR	(52)
2RUK	41–62	p62 subunit of TFIIH	NMR	(53)
6XRE	12–61	RNA polymerase II assembly	Cryo-EM	(54)
**c-MYC**				
7LQT	13–30	PNUTS	NMR	(55)
1MV0	55–68	BIN1	NMR	(56)
6E16	96–111	TBP + TAF1	X-ray	(57)
6E24	96–107	TBP + TAF1	X-ray	(57)
7T1Z	51–62	FBW7	X-ray	(58)
7T1Y	241–248	FBW7	X-ray	(58)

^a^This range corresponds to the residues that are resolved in the structure.

^b^Experimental method used to solve the structures.

X-ray, X-ray crystallography; NMR, nuclear magnetic resonance spectroscopy; cryo-EM, cryogenic electron microscopy.

### p53

**Fig. 3.**
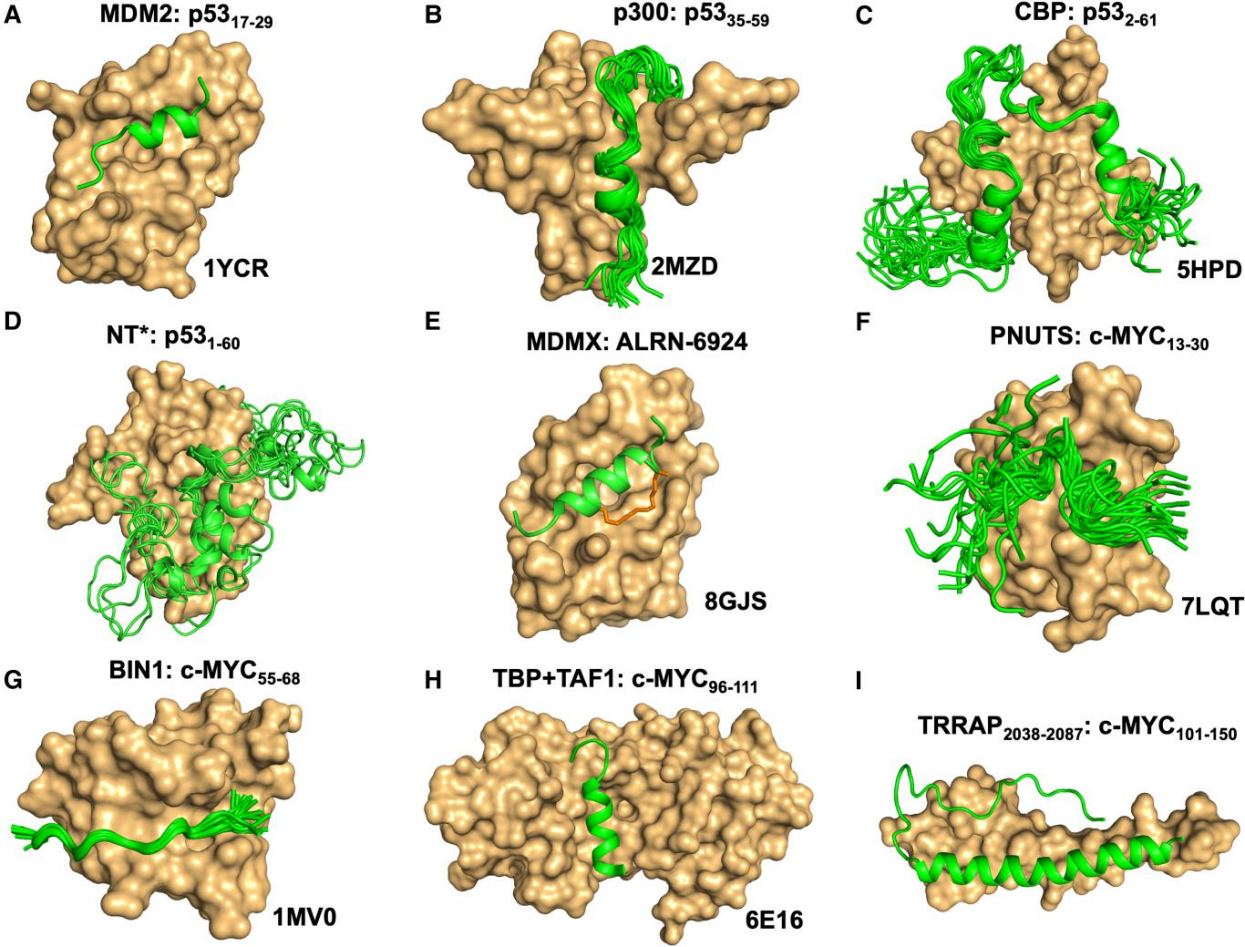
TAD interactions of p53 and c-MYC. A)–C) Experimentally determined structures of the p53 TAD with folded domains from MDM2, p300, and CBP, respectively. D) Ensemble of MD-simulated structures of p53 TAD with an engineered domain from spider silk protein (NT*). Material from Kaldmäe et al. (59). E) Crystal structure of p53 TAD1-based stapled-peptide derivative in complex with MDMX. The covalent linker forming the staple is shown in stick representation. F)–H) Experimentally determined structures of the c-MYC TAD with folded domains from PNUTS, BIN1, or a binary complex of TBP + TAF1, respectively. I) Integrated AlphaFold and MD-simulated complex structure of peptides from TRRAP with c-MYC TAD. Material from Lama et al. (60). The PDB IDs of all the structures obtained from experiments, along with the name of the binding partners and the residue range of p53/c-MYC TAD, are indicated. All the TAD structure ensemble in the complex is shown for structures solved with NMR. The TAD-binding partners are displayed in surface rendering, while the TADs are shown in cartoon representation.

The TAD1 of p53 is recognized by the murine double minute 2 (MDM2) protein, an E3 ubiquitin ligase that tags p53 for ubiquitination and subsequent proteasomal degradation (11). Crystal structures of the folded domain of MDM2 in complex with a 13-residue segment (residues 17–29) from the p53 TAD1 revealed that the peptide docks into a hydrophobic cleft on MDM2 and adopts a helical conformation (Fig. 3A). The specificity of the complex formation is driven primarily by a triad of p53 amino acids (F19, W23, and L26), which inserts into the MDM2 pocket and induces a helical structure in the almost entirely disordered TAD1 (43). A solution state structure of TAD2 from p53 has been reported in complex with the TAZ2 domain of histone acetyltransferase p300, an enzyme that stabilizes p53 and promotes local unwinding of the chromatin for gene transcription (48). The complex determined using NMR spectroscopy revealed that TAD2 formed an α-helix between residues 47 and 55 in the bound state (Fig. 3B).

The structure of the full-length p53 TAD (TAD1 + TAD2) has also been solved using NMR in complex with the TAZ2 domain of CREB-binding protein (CBP), which is a paralog of p300 (50). Here, the p53 TAD interacts in a bipartite manner with two interaction motifs that fold into helical segments upon binding TAZ2. The intervening regions between these helical motifs are unstructured and flexible (Fig. 3C). We recently showed that fusion of p53 to an engineered folded domain from a spider silk protein (termed NT*) resulted in increased expression and stability compared with wt p53 (59). Structural characterization using NMR and molecular dynamics (MD) simulations indicated the formation of a fuzzy complex where the TAD from p53 bound to a hydrophobic patch in the NT* domain through multiple nonspecific interactions (Fig. 3D). Interestingly, trapping the TAD in this manner resulted in stronger induction of p53 downstream targets, indicating that the transient, fuzzy interaction is sufficient to distort the finely tuned MDM2–p53 axis.

These complexes demonstrate the versatility provided by the disordered nature of p53 TAD to recognize diverse binding partners. The availability of high-resolution structural information has enabled the development of small molecule antagonist such as Nutlin and AMG232 that disrupt interaction between p53 and MDM2 (61). These molecules occupy the p53 TAD1 binding pocket on MDM2 and hence promote p53 stabilization by preventing its ubiquitination and degradation (62). Further, the p53–MDM2 structure was used to design and develop the first-in-class “stapled-peptide” compound (ALRN-6924) and a dual inhibitor of MDM2 and MDMX (a MDM2 homolog) to enter clinical trials (63, 64). Stapled peptides mimic the bound conformation of p53 TAD1 (Fig. 3E) through nonnative covalent linkers between the sidechains of amino acids at rationally selected positions in the peptide sequence (64–66). Such modifications have been effective in stabilizing the active helical conformation of the disordered TAD1 peptide in solution (64–66). Trapping the peptide in this manner reduces the entropic cost of recognition which the disordered TAD1 requires while undergoing structural remodeling in the process of binding MDM2/MDMX.

### c-MYC

Like p53, peptide derivatives from different regions of c-MYC TAD have been subjected to structure determination in complex with their binding partners. Protein phosphatase 1, a serine threonine phosphatase, and its regulatory subunit PNUTS are required to control the phosphorylation of c-MYC and thereby maintain its stability and occupancy at the chromatin (67). A peptide (residues 13–30) from the highly conserved MB0 motif present at the TAD of c-MYC has been shown to directly interact with the folded N-terminal domain (PAD) of PNUTS (55). The molecular structure solved by NMR showed that the disordered peptide anchors into the binding pocket of PAD and adopts multiple conformations with features of a fuzzy complex (Fig. 3F).

The bridging interactor 1 (BIN1) protein is a nucleocytoplasmic adaptor protein with tumor-suppressive features, and its interaction with c-MYC can modulate c-MYC-mediated transformation and transactivation (68). The BIN1:c-MYC recognition is dependent on the highly conserved MBI motif in the TAD of c-MYC. The structural characteristic of this interaction is provided by a proline-rich peptide fragment (residues 55–68), which form part of the MBI motif in complex with the C-terminal Src homology 3 domain of BIN1 (56). The structure determined using NMR showed that the c-MYC TAD peptide adopts a predominantly polyproline II secondary structure in the bound state (Fig. 3G).

The TATA-binding protein (TBP) is an essential component of the multimeric transcription factor IID (TFIID) initiation complex, which is responsible for RNA polymerase II assembly and gene expression (69). The structure of a c-MYC peptide derivative (residues 96–111), which is proximal to the conserved MBII motif in c-MYC TAD, has been reported in a ternary complex with TBP and the amino-terminal domain 1 of TBP-associated factor 1 (TAF1) protein (57). The structure solved using X-ray crystallography indicates that the c-MYC peptide docks into the grove formed by TBP + TAF1 binary complex and adopts an amphipathic helical structure (Fig. 3H).

For MBII, which is the most prevalent site for protein recruitment in c-MYC TAD, no structure of the motif with a target protein has been solved to date. The transformation/transcription domain–associated protein (TRRAP) is a critical cofactor of c-MYC and one of the biophysically well-characterized MBII-dependent binders (70, 71). We have recently shown using native mass spectrometry that peptide derivatives from c-MYC (residues 101–150, which harbors the MBII motif) and TRRAP (residues 2038–2087) form a 1:1 complex, suggesting specific association (60). Further, a modeled structure developed through a combination of AlphaFold and MD simulations suggested an elongated and predominantly helical conformation of the two peptides in the complex state (Fig. 3I). This model corroborates an earlier study which indicated that the interacting peptides from c-MYC and TRRAP undergo a “disorder-to-order” transition in the formation of a structurally stable conformation with high alpha helical character (71).

## Targeting TADs through conformational modulation

The structures described above show that the different peptide derivatives from the TADs of p53 and c-MYC adopt multiple conformations in their bound state, underlying their plasticity to recruit diverse binding partners. Importantly, the structures also suggest that molecules that can bind to these TADs have the potential to inhibit their interactions. Table 2 highlights a list of ligands that are reported to target the TAD of p53, c-MYC, and AR. It contains information on the name of the compounds, chemical structure, and the biophysical method used to validate TAD binding, as well as the latest stage of their development. The initial hit identification of such compounds does not have to accurately match the specific sequence or structural features of the binding interfaces. This is illustrated by the fact that some naturally occurring polyphenols such as epigallocatechin gallate (EGCG) or curcumin and their derivatives are potent aggregation inhibitors for IDPs (81–83). These molecules combine aromatic moieties and hydroxyl groups in a flexible scaffold, to engage in multiple nonspecific interactions involving hydrophobic contacts and hydrogen bonds, which blocks the sites required for self-association (81–83). The extension of such an approach in the context of targeting TAD by modulating their conformational flexibility is demonstrated with current examples from p53, c-MYC, and AR in this section.

**Table 2.** TAD inhibitors of p53, c-MYC, and AR.

Compound	Structure	Target	Method^a^	Evaluation	References
EGCG	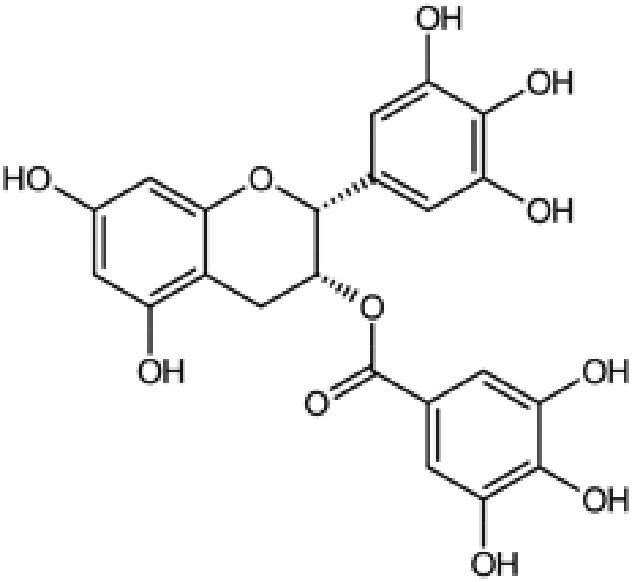	p53	SPR	Inhibition of PPI *in vitro*	(60, 72)
		c-MYC	MS		
PKUMDL-RH-1047	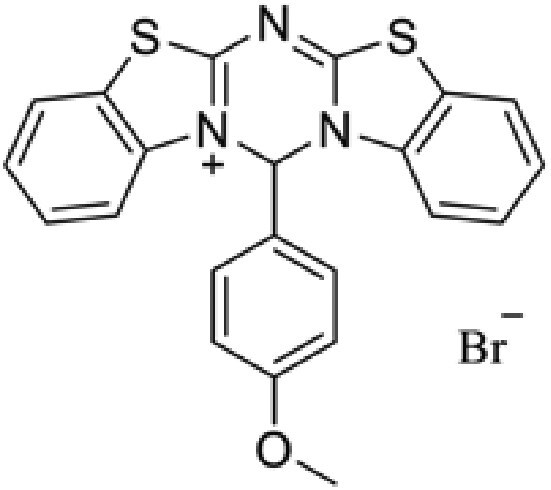	p53	SPR	Inhibition of PPI *in vitro*	(73)
EN4^b^	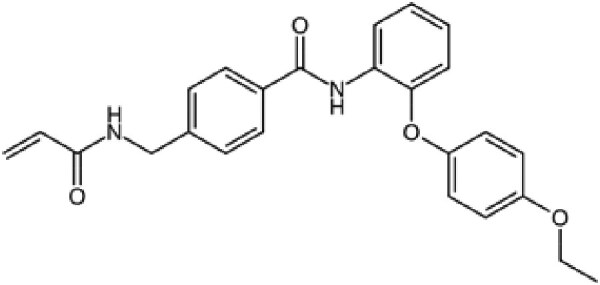	c-MYC	MS	Inhibition of transcriptional activity *in vitro*	(74)
EPI-001	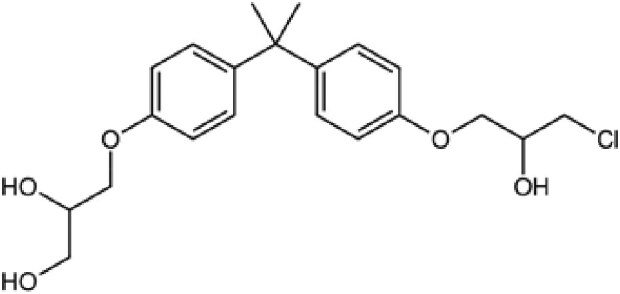	AR	NMR	Derivatives	(75)
				EPI-506 and	NCT02606123
				EPI-7386 tested in phase I clinical trials	NCT04421222
UT-143					
	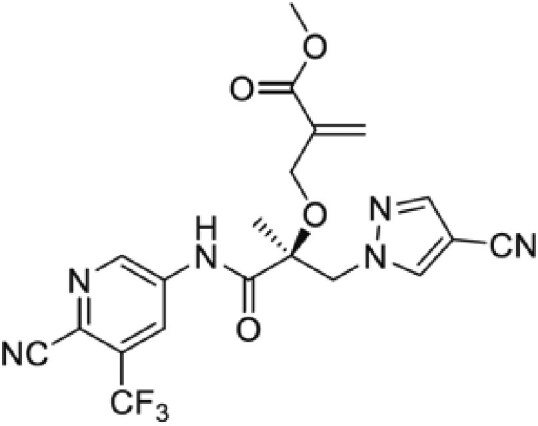	AR	MS	Inhibition of tumor growth *in vivo*	(76)
QW07	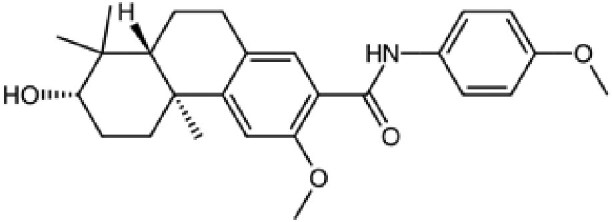	AR	SPR	Inhibition of tumor growth *in vivo*	(77)
SC-428	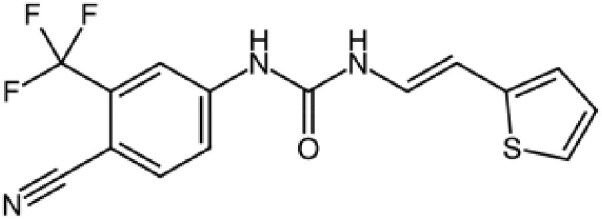	AR	SPR	Inhibition of tumor growth *in vivo*	(78)
ET-516	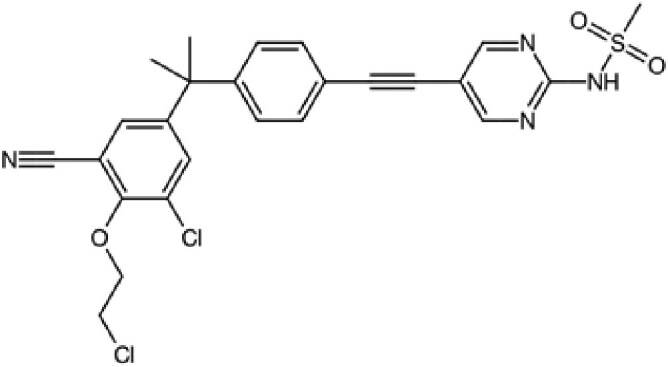	AR	MST	Inhibition of tumor growth *in vivo*	(79)
ASR-600	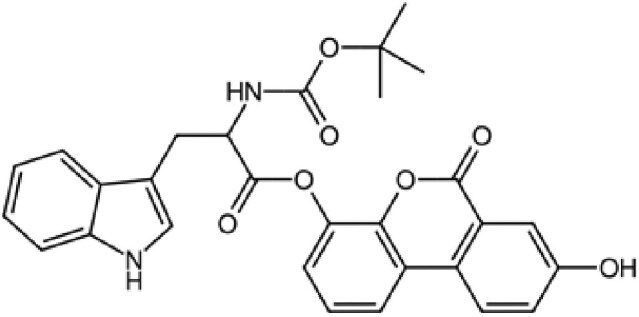	AR	STD NMR	Inhibition of tumor growth *in vivo*	(80)

^a^Biophysical method used to validate binding.

^b^This compound is shown to covalently interact with C171, which is slightly outside the residue range of c-MYC TAD defined in this review.

SPR, surface plasmon resonance; MS, mass spectrometry; NMR, nuclear magnetic resonance spectroscopy; MST, microscale thermophoresis; STD NMR, saturation transfer difference nuclear magnetic resonance spectroscopy; PPI, protein-protein interaction.

### p53

**Fig. 4.**
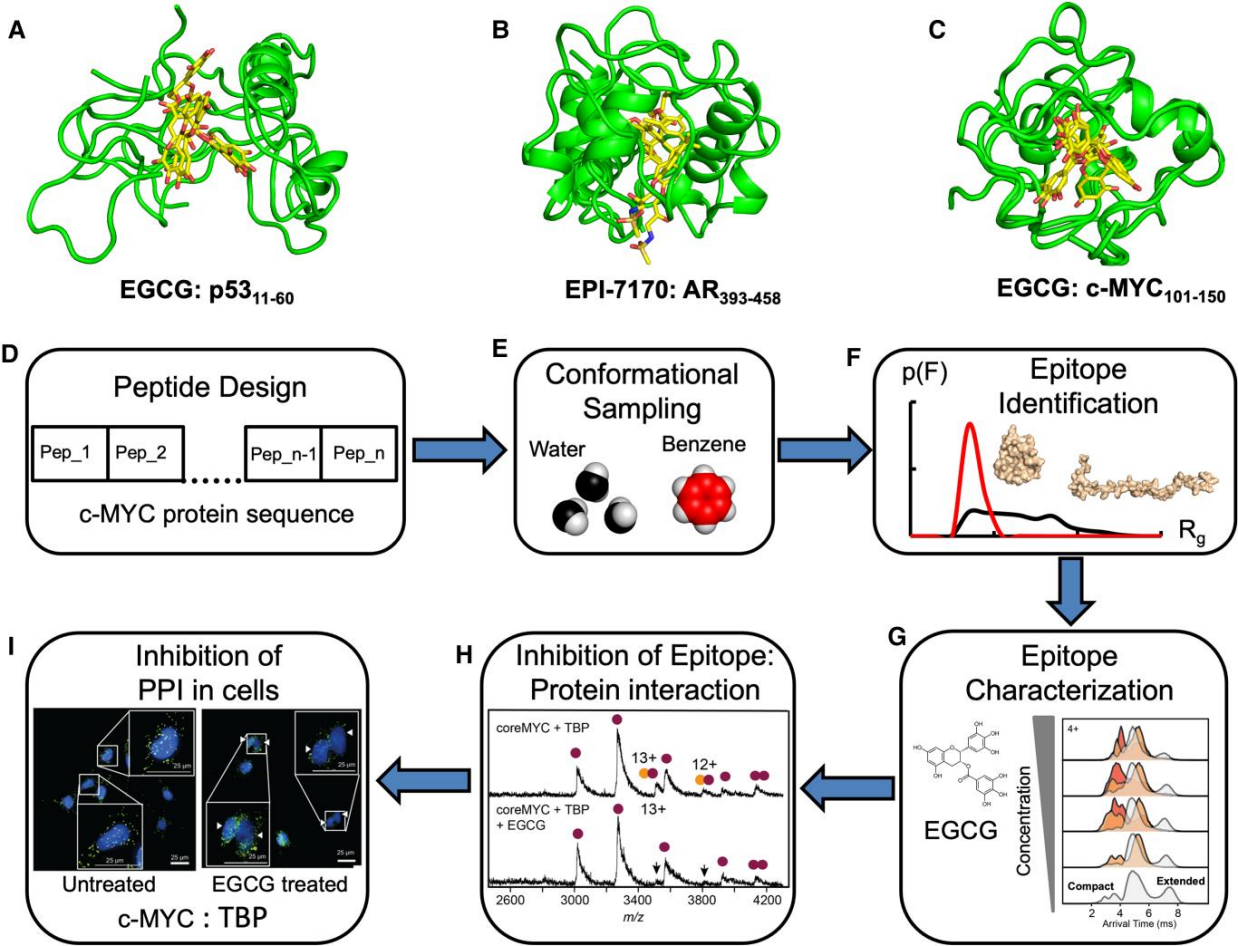
Inducing the compact conformation of TAD. A)–C) Ensemble of MD-simulated structures of peptide segments from TAD of p53, AR, and c-MYC, respectively, with EGCG (p53 and c-MYC) and EPI-7170 (AR). The TAD segments are shown in cartoon and the small molecules in stick representations. The residue range of the TAD used in the MD simulations is indicated. D)–I) Schematic representation of the pipeline developed for identification and characterization of an epitope within c-MYC that can undergo ligand-inducible conformational modulation. D) Representation of the different peptide derivatives from c-MYC, E) MD simulations of the peptides with and without benzene, F) conformational distribution of coreMYC as a function of the radius of gyration, G) arrival time distribution of coreMYC from ion mobility mass spectrometry of the peptide with increasing concentrations of EGCG, H) native mass spectra of coreMYC + TBP with and without EGCG, and I) representative images from proximity ligation assay of c-MYC and TBP interaction in cells with and without the treatment of EGCG. The specific details of the individual steps in the pipeline are also highlighted in each panel. Material from Lama et al. (60).

A recent study by Zhao et al. (72) showed that EGCG interacted with the N-terminal disordered domain of p53 with binding affinity in the low micromolar range using surface plasmon resonance (SPR). NMR measurements showed that the TAD1 and TAD2 were particularly responsive to EGCG binding. The study used analytical ultracentrifugation, small-angle X-ray scattering, and MD simulations to demonstrate that EGCG interaction with the disordered domain was dynamic and that the small molecule induced a compact subpopulation of the p53 TAD. We performed an independent MD simulation of 50 amino acid (residues 11–60) peptide segment from p53 TAD with EGCG and observed that it could indeed capture the derivative in a compact conformation (Fig. 4A). Zhao et al. (72) have further reported that EGCG could disrupt the p53–MDM2 interaction and prevent ubiquitination of p53 in an in vitro ubiquitination assay, which correlates with reduced p53 degradation and stability in cells treated with EGCG. This study adds to the growing evidence that small molecules can associate and effectively promote the conformational modulation of disordered regions to prevent protein–protein interaction.

### AR

One of the most encouraging therapeutic developments in the direct targeting of disordered regions with small molecule compounds has been against the N-terminal TAD of AR (84, 85). Different families of compounds have been characterized to interact directly with the activation domain, and the majority of them have also been shown to inhibit xenografts of different prostate cancer models (Table 2) (22, 86, 87). A couple of derivatives from the EPI series (EPI-506 and EPI-7386) were in fact subjected to clinical trials for the treatment of castration-resistant prostate cancer (22, 88). Although both trials were eventually suspended, the advancement into clinical evaluation indicates the potential for targeting the disordered TAD in AR as a therapeutic strategy. These prodrugs were derived from the first (EPI-002) and second (EPI-7170) generations of bisphenol-A scaffold-based AR-TAD inhibitors, respectively. Characterization of EPI-002 interaction with AR-TAD using NMR indicated that the largest chemical shift perturbations were localized to the TAU-5 transactivation unit of AR-TAD (75). Further exploration using MD simulations revealed that both EPI-002 and EPI-7170 sampled a wide range of binding configurations, which induced the formation of partially folded compact states in TAU-5 (89). However, EPI-7170 was found to form elevated intermolecular interactions in the bound ensemble that stabilized the collapsed conformation more potently and hence displayed higher affinity to TAU-5 than EPI-002 (Fig. 4B). This study thus provided a rationale for the mechanism of inhibition and the experimentally observed differences in the binding potency of the two compounds. As in the case for p53, compaction of the molecular recognition elements in the TAD of AR is the primary mechanism of inhibition for these therapeutic small molecules, which prevents interactions between AR and the transcriptional machinery. AR is also reported to undergo liquid–liquid phase separation (LLPS) to form transcriptionally active biomolecular condensates (90). The various domains in AR cooperate to facilitate effective phase separation, with TAD playing the predominant role (79, 90, 91). Importantly, ligands that target AR TAD and reduce LLPS also suppress AR transcriptional activity and inhibit tumor growth *in vivo* (79, 92).

### c-MYC

We have recently reported a proof-of-concept study in which we developed a pipeline that led to the identification of ligand-inducible conformational switch in the TAD of c-MYC (Fig. 4C) (60). The first part of the process involved computational peptide screening using probe-based MD simulations across the entire disordered c-MYC protein to find segments whose structural ensemble can be modulated with exogenous ligands (Fig. 4D). A set of 17 50-residue peptide derivatives were designed and subjected to MD simulations with and without benzene as the molecular probe (Fig. 4E). The conformational sampling from these simulations highlighted that the impact on the ensemble distribution is most substantial for the peptide spanning residues 101–150, which includes the conserved MBII motif of c-MYC. This epitope (termed coreMYC) that samples both compact and extended states in water undergoes a shift towards a predominantly compact conformation with the addition of benzene (Fig. 4F). We found that the epitope is the most hydrophobic region of c-MYC composed predominantly of bulky nonpolar residues, which makes it particularly sensitive to conformational modulation by benzene. Extending the computational approach to small molecules, we found that the polyphenolic compound EGCG similarly induced compaction of the epitope.

The second part of the pipeline included experimental examination of the c-MYC:EGCG complex using native ion mobility mass spectrometry (Fig. 4G). These experiments showed that the small molecule interacted with the peptide in a nonspecific manner driving the conformational equilibrium towards a predominantly compact state. This conformational switch inhibited the interaction of the epitope in the TAD with one of its binding partners TBP (Fig. 4H). The inhibitory effect was also observed in cancer cells where treatment with EGCG reduced binding of c-MYC to TBP (Fig. 4I), but not to its obligate partner MAX, which binds to the C terminus of c-MYC (60). Significant efforts have been made on disrupting the c-MYC:MAX heterodimerization by targeting the basic-helix-loop-helix leucine zipper (bHLHZip) domain, involved in this association (93, 94). Comparatively, our survey of the literature indicates that direct targeting of the N-terminal domain to inhibit c-MYC is relatively scarce (Table 2). Therefore, the study (60) provides an impetus in this direction and presents a blueprint for the systematic identification and characterization of ligand-binding interfaces in IDPs. It further emphasizes that shape-shifting compounds could be the hallmark for directly targeting disordered regions like the TAD of c-MYC.

## Outlook

Disordered regions such as the TAD of transcription factors exert their biological functions without a need for well-defined structures. Instead, they rely on occupying an ensemble of states that facilitates fuzzy complexes, conformational selection, induced folding, or their combination for binding partner recognition. However, their intrinsic flexibility and the lack of targetable sites often prevent the utilization of conventional methods to target disordered proteins to combat disease. Instead, modulation of their conformational landscape has emerged as a potential targeting mechanism. More specifically, shifting the equilibrium between the different conformational states that an IDP can occupy may prevent it from accessing functionally important conformations. For example, molecular probes can be utilized to bind and trap the disordered protein in a nonactive conformational state. This possibility is highlighted here using three well-established anticancer targets p53, c-MYC, and AR. Collectively, recent efforts show that direct interaction of small molecules with the disordered TADs in these proteins shifts their conformation towards a compact state and inhibits protein–protein interaction. Through the collaborative application of complimentary strategies, we can systematically explore the dynamic landscape of disordered proteins, with the goal of identifying innovative ways and promising compounds to target their activity for therapeutics.

## Data Availability

There are no data underlying this work.
